# Rules for Practicing Iodine Injections in Ascites

**Published:** 1855-03

**Authors:** 


Rules for practising Iodine Injections in Ascites. By M. Tessier.—These rules are three in number:—

1st. Not to empty the peritoneal cavity before practising the injection. This is obvious, for if the injection be not diluted, and diffused by the ascitic fluid, it might act too powerfully and partially upon the peritoneum. Death from peritonitis has happened from the want of attention to this precaution.

2d. To order the strength of the injection in conformity to the composition of the peritoneal fluid, the strength being in direct relation to the alkalinity and albuminosity. When this liquid is clear and but slightly alkaline and albuminous, M. Tessier injects from twenty to thirty grammes of tincture of iodine, and two grammes of iodide of potassium; where the liquid is decidedly albuminous, sanguineous or purulent, and especially if it be very alkaline, he doubles these quantities.

3d. To practise a preliminary tapping some day previously, if the abdomen is very voluminous, in order to diminish the peritoneal surface, and so lessen the risk of peritonitis.—*Medical Gazette.*

